# Dextran-Based Injectable Hydrogel Composites for Bone Regeneration

**DOI:** 10.3390/polym15234501

**Published:** 2023-11-23

**Authors:** Patrícia Alves, Ana Filipa Simão, Mariana F. P. Graça, Marcos J. Mariz, Ilídio J. Correia, Paula Ferreira

**Affiliations:** 1 University of Coimbra, CIEPQPF, Department of Chemical Engineering, Faculty of Sciences and Technology, Rua Sílvio Lima, 3030-790 Coimbra, Portugal; gouveia.fii@gmail.com (A.F.S.); mmariz@eq.uc.pt (M.J.M.); icorreia@fcsaude.ubi.pt (I.J.C.); paula.ferreira@ipc.pt (P.F.); 2 CICS-UBI, Health Sciences Research Centre, Faculty of Health Sciences, University of Beira Interior, 6200-506 Covilhã, Portugal; marianaf_g@hotmail.com; 3 Applied Research Institute, Polytechnic Institute of Coimbra, Rua da Misericórdia, Lagar dos Cortiços—S. Martinho do Bispo, 3045-093 Coimbra, Portugal

**Keywords:** oxidized dextran, injectable hydrogels, bone regeneration, drug release

## Abstract

Currently, bone infections caused by diseases or injuries are a major health issue. In addition, the conventional therapeutic approaches used to treat bone diseases or injuries present several drawbacks. In the area of tissue engineering, researchers have been developing new alternative therapeutic approaches, such as scaffolds, to promote the regeneration of injured tissues. Despite the advantages of these materials, most of them require an invasive surgical procedure. To overcome these problems, the main focus of this work was to develop scaffolds for bone regeneration, which can be applied using injectable hydrogels that circumvent the use of invasive procedures, while allowing for bone regeneration. Throughout this work, injectable hydrogels were developed based on a natural polymer, dextran, along with the use of two inorganic compounds, calcium β-triphosphate and nanohydroxyapatite, that aimed to reinforce the mechanical properties of the 3D mesh. The materials were chemically characterized considering the requirements for the intended application: the swelling capacity was evaluated, the degradation rate in a simulated physiological environment was assessed, and compression tests were performed. Furthermore, vancomycin was incorporated into the polymeric matrices to obtain scaffolds with antibacterial performance, and their drug release profile was assessed. The cytotoxic profile of the hydrogels was assessed by an MTS assay, using osteoblasts as model cells. The data obtained demonstrated that dextran-based hydrogels were successfully synthesized, with a drug release profile with an initial burst between 50 and 80% of the drug. The hydrogels possess fair biocompatibility. The swelling capacity showed that the stability of the samples and their degradation profile is compatible with the average time period required for bone regeneration (usually about one month) and have a favorable Young’s modulus (200–300 kPa). The obtained hydrogels are well-suited for bone regeneration applications such as infections that occur during implantation or bone graft substitutes with antibiotics.

## 1. Introduction

Bone fractures associated with diseases (cancer, osteomyelitis) or injuries (fractures) are a major health problem. Up to now, bone autografts and allografts, as well as the use of implants (to replace defective organs or tissues) and prostheses (mostly designed to replace a missing part of the body) have been used to manage these health conditions. Despite the recent achievements in this area of knowledge, these therapeutic approaches have several flaws, such as the lack of donors, risk of infections, death of surrounding tissues, rejection of implants, and ultimately, the death of patients. In addition, these therapeutic approaches produce pain and discomfort for patients. Moreover, all these techniques require invasive surgical procedures [1].

Over the last decades, tissue engineering (TE) is a scientific area of intervention which has been increasingly recognized in the field of medicine. Recent studies have sought to overcome the limitations of conventional therapies, allowing for less discomfort for the patient and also to reduce the risks for the patient [2,3]. Within the area of TE, scaffolds (three-dimensional support matrices), pre-formed or injectable, have been developed to be used for bone regeneration. These 3D structures are intended to become therapeutic alternatives to some classic approaches, due to their ability to mimic the native bone structure, thus promoting the regeneration of the injured tissue; at the same time, these structures might also allow for the incorporation of bioactive substances into the scaffold matrix [4]. In this way, the development of injectable scaffolds, which allow for the incorporation of active substances, becomes promising [5,6], establishing an alternative to implants and grafts, and overcoming the problems of the lack of bone donors, the rejection of prostheses, and their associated effects. The use of these 3D structures contributes to making chirurgical procedures less invasive, thus making the recovery process less painful, reducing the risk of infection, and improving the aesthetic aspect of the wound. In addition, it is also a versatile alternative, as injectable hydrogel scaffolds adapt to the lesion, assuming the morphology of the defect, despite its shape. It also allows for an easy incorporation of active substances during the preparation of the scaffolds, and reduces the risk of infections [7].

**Figure 1:**
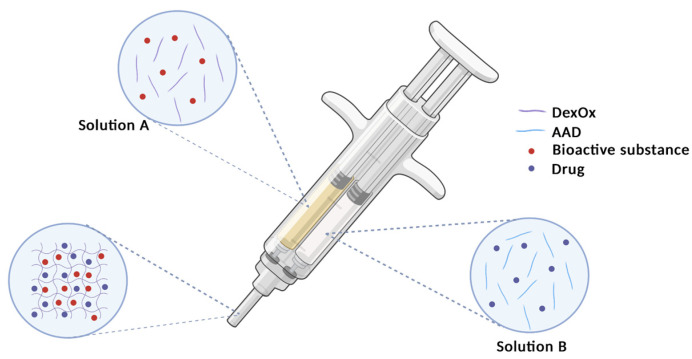
Schematic representation of a two-tube syringe for the application of the hydrogels.

The present work focused on the production of dextran-based materials for bone tissue filling and regeneration. Dextran is a polysaccharide and therefore a natural polymer that provides some inherent advantages, namely its biodegradability and biocompatibility. Therefore, this polymeric material has been previously applied in the preparation of in situ injectable hydrogels; it is able to fill and adapt its shape to the bone defects prior to crosslinking reactions. These hydrogels have proven to provide suitable extracellular matrix conditions [8] and their in vitro capacity to support the attachment and proliferation of cells has been previously assessed [9]. The reported results indicate that cells were unable to spread on the surfaces of dextran hydrogels, and rather proliferated as clusters, showing that native dextran exhibits poor cell adhesive properties [10,11]. Therefore, some strategies may be followed in order to increase the material’s bioactivity. One of these approaches is the inclusion of inorganic compounds (e.g., hydroxyapatite [12], β-tricalcium phosphate [13], or bioactive glass [14]) on the polymeric matrix. Another advantage of the incorporation of these inorganic materials into the hydrogels’ structure is the reinforcement of their mechanical properties and therefore the improvement of their performance. Moreover, hydrogels may be used as a support for suitable drugs that may assist in the processes of healing and tissue regeneration. In order to achieve the maximum potential of this approach, a two-tube syringe is required so that the prepared solutions are mixed at the time of injection, allowing gelation to occur at the desired location. Figure 1 represents the operation scheme of this syringe, in which solution A represents DexOx and the bioactive substance, and solution B represents the crosslinker and the drug. The set of components A and B is represented on the needle of the syringe, since their mixture takes place before the fluid leaves the syringe, providing better homogeneity in the hydrogel.

In this work, dextran was modified, through the introduction of aldehyde groups, to allow its subsequent crosslinking, and the formation of the hydrogel. In addition, these hydrogels allow the incorporation of drugs into their matrix, which aids in the healing process of the lesion, avoiding infectious processes to occur. In the present work, the incorporated drug was vancomycin, which is a bactericidal antibiotic. Moreover, the incorporation of inorganic materials was also tested to improve the mechanical properties of the hydrogel. Two different inorganic compounds (hydroxyapatite and β-tricalcium phosphate) were tested in order to compare the obtained results and evaluate the individual performance of each material. Therefore, the contribution of each inorganic compound to the overall performance of the scaffolds was evaluated.

The developed materials were characterized to evaluate the necessary requirements for the intended application. ATR-FTIR and ^1^H NMR analyses were performed to chemically characterize the materials and to verify the chemical modification of the polymers. The hydrogels’ swelling ratio, biodegradability, and drug delivery at different pH values (2; 5; 7.4; and 9) were determined. Moreover, compression tests were performed to assess their mechanical properties. Finally, the hydrogels’ biocompatibility was evaluated by incubating samples previously prepared with each inorganic compound with normal human osteoblast cells and by assessing cellular viability.

## 2. Materials and Methods

### 2.1. Materials

The following materials were used in the different stages of the formulation of the scaffolds: Dextran (70,000, TCI, Odivelas, Portugal), sodium m-periodate (99%, Fisher Scientific, London, UK), trinitrobenzenosulfonic acid (TNBS, Sigma-Aldrich, Sintra, Portugal), trichloroacetic acid (≥99.5%, Sigma-Aldrich, Portugal), tert-butyl carbazate (>97%, TCI, Zwijndrecht, Belgium), diethylene glycol (>99%, Chem-Lab, Zedelgem, Belgium), adipic acid dihydrazide (AAD > 99%, TCI, Zwijndrecht, Belgium), dialysis membranes (12–14,000 Da, Medicell Membranes Ltd., London, UK), phosphate buffered saline (PBS, Panreac, Cascais, Portugal), potassium chloride (KCl, ≥99.5%, Sigma-Aldrich, Sintra, Portugal), Vancomycin (Hikma Pharmaceuticals USA Inc., Columbus, OH, USA), β-tricalcium phosphate (β-TCP, gently given by Artur Salgado SA, Porto, Portugal), Nanohydroxyapatite (nHAp, gently given by Artur Salgado SA, Porto, Portugal), fetal bovine serum free from antibiotics (FBS, Biochrom AG, Berlin, Germany), cell culture plates (Thermo Fisher Scientific, Porto, Portugal), T-flasks (Thermo Fisher Scientific, Porto, Portugal), 3-(4,5-dimethylthiazol-2-yl)-5-(3-carboxymethoxyphenyl)-2-(4-sulfophenyl)-2H-tetrazolium (MTS, Promega, Madison, WI, USA), Dulbecco’s modified eagle’s medium F-12 (DMEM-F12, Sigma-Aldrich, Sintra, Portugal), trypsin (Sigma-Aldrich, Sintra, Portugal), and normal human osteoblast (hOB, Cell Applications, Inc., San Diego, CA, USA).

### 2.2. Synthesis of Oxidized Dextran (DexOx)

**Figure 2:**
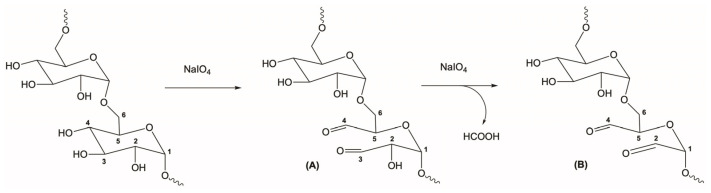
Representative scheme of the Dex oxidation through reaction with sodium periodate. (**A**) First oxidation, introduction of aldehyde groups at C3 and C4. (**B**) Second oxidation, introduction of the aldehyde group at C2 and release of formic acid.

The procedure used to obtain the oxidized dextran (DexOx) was based on previous works [15,16]. Briefly, dextran was reacted with sodium periodate, in order to introduce the aldehyde functional groups, for the subsequent synthesis of the hydrogels. This step started with the preparation of an aqueous solution of dextran, where 2 g of dextran were dissolved in 28 mL of ultrapure water. Next, an aqueous solution of the oxidizing agent was prepared by dissolving sodium periodate in ultrapure water in the proportions reported in the literature to obtain DexOx with oxidation degrees (OD) of 25% and 50% [17]. The sodium periodate solution was added dropwise to the dextran solution for 20 min and allowed to react for 24 h at room temperature in the absence of light. Then, diethylene glycol was added to the reaction medium, in an equimolar amount, in order to stop the reaction. DexOx was then obtained as a product of the reaction (Figure 2).

Afterwards, DexOx was purified by dialysis against water for 3 days, changing the water 2 to 3 times a day, in order to remove the by-product from the second oxidation of dextran (formic acid), impurities, traces of unreacted sodium periodate, and diethylene glycol. Finally, the samples were lyophilized.

### 2.3. Synthesis of DexOx/AAD Scaffolds

After the introduction of the aldehyde groups into the dextran structure, they can be crosslinked with AAD. For that purpose, a solution of DexOx (20% (*w*/*v*)) in PBS (pH 7.4) was prepared and placed in a bath at 37 °C until complete dissolution. Next, a solution of the crosslinking agent, AAD, was prepared (10% and 20% on a molar basis, taking into account the number of glucose residues in the dextran). This concentration was determined according to Equation (1):

(1) $$\left[AAD\right]=\frac{\frac{m_{DexOx}}{M_{w_{ResGlu}}}\times 0.1M_{w_{AAD}}}{V}$$

where $M_{w_{Res\;Glu}}$ is the molar mass of the glucose residues in the original dextran, $m_{DexOx}$ is the mass used in the DexOx solution, $M_{w_{AAD}}$ is the molar mass of AAD, and V is the volume of distilled water used to prepare the DexOx solution. Then, 250 μL of DexOx solution were transferred to a 24-well plate, and 250 μL of the solution of AAD were mixed, allowing them to react overnight at 37 °C [15].

In addition, 1% (*w*/*v*) inorganic compounds (β-TCP or nHAp), were also mixed in the DexOx (OD = 50%) solution. Figure S1 shows an image of the obtained hydrogels and Table 1 summarizes their compositions, varying the amount of the crosslinker and the presence of inorganic compounds, in order to assess their influence on the final properties of the prepared materials.

### 2.4. Drug Incorporation

**Figure 3:**
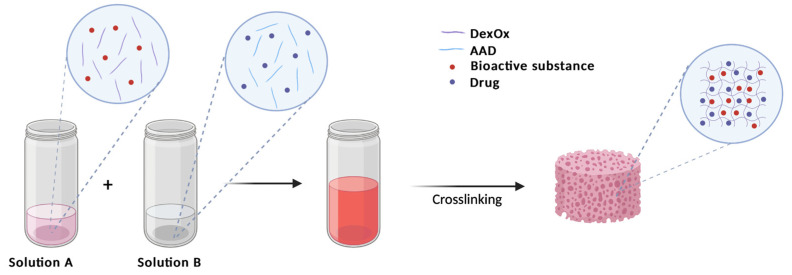
Schematic representation of drug incorporation into the preparation scaffold.

Vancomycin was selected to be incorporated within the scaffold since it is a drug that is widely used to treat bone infections. According to the application intended for the produced hydrogels, the incorporation of the drug must be carried out before the gelling step, since this occurs in situ. Thus, to incorporate the drug into the material, the entrapment method was chosen, which is based on introducing the drug into the solutions that will subsequently produce the hydrogels (Figure 3). It has been suggested that the minimum dose of this antibiotic should be 2 g per 40 g of scaffold [18]. For that purpose, a 5% (*w*/*v*) vancomycin aqueous solution was added to the AAD solution, which was then added to the DexOx solution according to the compositions in Table 1.

### 2.5. Characterization

#### 2.5.1. Attenuated Total Reflectance-Fourier Transform Infrared Spectroscopy (ATR-FTIR)

ATR-FTIR analyses were performed using a Bruker Tensor 27 spectrophotometer (Buker Optik GMGH, Ettlingen, Germany). The spectra obtained were performed with 64 scans, with a resolution of 400 cm^−1^, in the spectrum range of 550–4000 cm^−1^. This technique was applied to the produced hydrogels, as well as to the chemical compounds used in their synthesis (dextran, DexOx, AAD, β-TCP, and nHAp).

#### 2.5.2. Nuclear Magnetic Resonance (^1^H NMR)

^1^H NMR spectra were obtained using a Bruker Avance III 400 MHz spectrometer. Deuterated water sample solutions (DexOx OD = 25% and OD = 50%) reacted with t were analyzed in tubes of 5 mm diameter, using tetramethylsilane (TMS) as an internal standard.

#### 2.5.3. Degradation

Fresh hydrogels were placed into buffer solutions with different pH values (2; 5; 7.4; and 9) at 37 °C, to evaluate the influence of pH on the degradation of the hydrogels. The hydrogels were removed from the buffers, excess liquid was removed from their surface, and they were weighed daily (*m*_*t*_). The buffers were replaced at each weighing. This process was carried out until the loss of the integrity of the hydrogels, that is, until their collection for weight registration was no longer possible due to their state of degradation.

#### 2.5.4. Swelling Ratio (Sr)

In order to evaluate the swelling capacity of the hydrogels after gelation, they were frozen and subsequently lyophilized. The lyophilization step is necessary, since during crosslinking of hydrogels, water is released and, therefore, at the end of gelation, the hydrogels present an initial water content that needs to be disregarded. Therefore, after being lyophilized, the hydrogels were weighed (*m*_*i*_) and placed in buffers at a temperature of 37 °C for 24 h to allow swelling, and then weighed (*m*_*f*_) again. In the human organism, pH variations occur according to health conditions and with the type of living tissues, among other factors. Thus, the hydrogels were placed in buffers with different pH values (2, 5, 7.4, and 9). The swelling ratio (*S*_*r*_) was calculated using Equation (2) [19].

(2) $$S_{r}=\frac{m_{f}-m_{i}}{m_{i}}$$

#### 2.5.5. Drug Release

The drug release profile was accessed by placing the fresh hydrogels, each one in a closed vial, with 10 mL of PBS (pH7.4) and incubated in an Excella E24 Incubator Shaker (from New Brunswick Scientific) at 37 °C under constant shaking at 100 rpm. The drug release profile was measured in a Jasco V-550 UV-Vis spectrophotometer, using the Fixed Wavelength Measurement technique. The relationship between absorbance and drug concentration was obtained by a calibration curve with vancomycin standard solutions. Measurements were performed at the following time periods: 1 h, 3 h, 6 h, 24 h, 48 h, 72 h, 7 d, 14 d, and 21 d. At each period, 1.5 mL of the supernatant liquid was withdrawn for analysis and replaced with fresh PBS (pH 7.4). Readings were performed at 280 nm [20,21].

#### 2.5.6. Compression Tests

Compression tests allowed for the determination of the elastic modulus/Young’s modulus, which provides information about the mechanical properties of the material. Compression tests were carried out using an Inspekt Mini Series, with a 3 kN load cell (Hegewald & Peschke), until failure (complete destruction of the sample). During these tests, fresh samples were subjected to a unidirectional (axial) force, caused by compression of the cell at a rate of 1 mm/min and with an initial force of approximately 2 N. All samples had a cylindrical shape with an average diameter of 14.22 ± 0.32 mm and an average thickness of 2.25 ± 0.22 mm.

#### 2.5.7. Cytotoxicity Assays

The cellular viability and cytotoxicity of the produced materials were evaluated using 3-(4,5-dimethylthiazol-2-yl)-5-(3-carboxymethoxyphenyl)-2-(4-sulfophenyl)-2H-tetrazolium (MTS) according to ISO 10993-5:2009 (Biological evaluation of medical devices—Part 5: Tests for in vitro cytotoxicity) [22]. Briefly, fresh hydrogel samples (n = 5, sizes inferior to 10% of the well’s area) were placed in 96-well plates and then sterilized by UV irradiation (254 nm, ≈7 mW/cm^2^) for 1 h. Since the material is intended for bone tissue applications, normal human osteoblast cells (hOB) were seeded at a density of 1 × 10^4^ cells/well into the wells containing the samples and incubated at 37 °C and 5% CO_2_ in a humidified atmosphere for 1, 3, and 7 days. As a positive control (K^+^), cells were incubated with EtOH (70%), and for the negative control (K^−^), cells were incubated with culture medium. After incubation, the medium of each well was replaced by 100 µL of fresh culture medium and 20 µL of MTS/phenazine methosulfate reagent solution and incubated for 4 h. After that, the optical density (OD) of each sample was measured using an absorbance microplate reader (Biorad xMark Microplate Spectrophotometer) at 490 nm. The cell viability was determined according to Equation (3):

(3) $$Cell\;Viability\;\left(\%\right)=\frac{{OD}_{t}}{{OD}_{c}}\times 100\%$$

where ${OD}_{t}$ is the optical density of the cells treated with the different samples, and ${OD}_{c}$ is the optical density of the non-treated control cells.

## 3. Results

### 3.1. Attenuated Total Reflectance-Fourier Transform Infrared Spectroscopy (ATR-FTIR)

**Figure 4:**
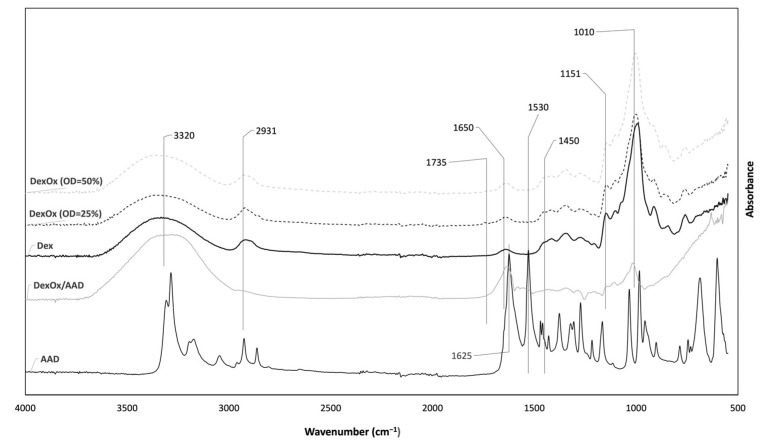
ATR-FTIR spectra of AAD, Dex, DexOx (OD = 25% and OD = 50%), and DexOx/AAD hydrogel.

Figure 4 shows the ATR-FTIR spectra of AAD, dextran, DexOx, and the DexOx/AAD hydrogel. The analysis of these spectra allows us not only to chemically characterize the samples through the identification of characteristic bonds, but also to verify whether the oxidation and cross-linking processes were successful.

From the Dex and DexOx spectra, it is difficult to identify significant differences corresponding to the introduction of aldehyde groups. According to the literature, the band corresponding to the stretching of the C=O bond (carbonyl) of the aldehyde groups would be visible at 1735 cm^−1^; however, this peak is not evident in the spectra. This is due to the low intensity of this peak for lower OD values; however, for an OD of 50%, it would already be possible to observe a more pronounced peak [15]. Nevertheless, in the DexOx spectrum (OD = 50%), the corresponding peak continued not to be observed. This fact may be related to the formation of hemiacetals between the aldehyde groups and the hydroxyl groups [23]. Analyzing the DexOx/AAD spectrum, a band at 1650 cm^−1^ concerning the stretching of the C=N bond of the hydrazone bonds can be seen, which confirms the crosslinking of DexOx with AAD [15]. The presence of this band allows us to indirectly conclude that the oxidation reaction of Dex took place, since crosslinking with AAD was only possible after the Dex oxidation. The AAD spectrum shows a peak at 1625 cm^−1^ that corresponds to the bending of the N-H bond from the amine group. After crosslinking, this peak should not be present in the DexOx/AAD spectrum; however, it cannot be clearly distinguished, since from the crosslinking, the peak at 1650 cm^−1^ shows a broad band in the same wavelength. The same happens with the peak at 1530 cm^−1^ in the AAD spectrum, from the bending of the N-H bond, and which does not appear due to the crosslinking and widening of the corresponding area [15].

The band at 1151 cm^−1^ is related to C-O-C bond vibrations and glycosidic bridges in Dex, DexOx, and DexOx/AAD. In the Dex and DexOx spectra, it is also possible to observe the bands at 2931 cm^−1^ and 1450 cm^−1^ assigned to the stretching of the C-H bond, the latter being less pronounced. The band at 1010 cm^−1^ appears due to the flexibility present in the α-1,6 glycosidic bonds [24].

At 3320 cm^−1^, the band corresponding to the OH group stretching of the polysaccharide segments is shown, both in the DexOx/AAD, DexOx, and Dex spectra [25]. In the DexOx/AAD hydrogel spectrum, this band appears with a wider shape, which may be related to the presence of some water in the Dex/AAD hydrogel network.

**Figure 5:**
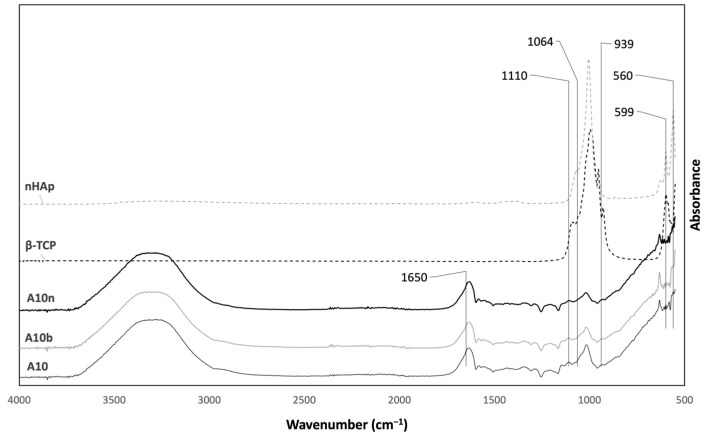
ATR-FTIR spectra of nHAp, β-TCP, and hydrogels with nHAp e β-TCP.

The spectra from the two inorganic compounds and the hydrogels with and without inorganic compounds can be seen in Figure 5.

The bands from 939 to 1110 cm^−1^ refer to the different vibrations of the PO_4_^3−^ ion. In addition, the bands at 560 cm^−1^ and 599 cm^−1^ correspond to the bending of the PO_4_^3−^ ion, which is present in the spectra of both inorganic compounds. The absorption peak located at 1064 cm^−1^ corresponds to the asymmetric stretching of the P-O bond [26]. Comparing the spectra of the hydrogels with and without the presence of inorganics, no differences are detected due to the low proportion of inorganic compounds introduced in the hydrogels. The band at 1650 cm^−1^ is also present in the spectra of the hydrogels DexOx/AAD/β-TCP, DexOx/AAD/nHAp, and DexOx/AAD, from the C=N bond stretching, due to the crosslinking reaction.

### 3.2. Nuclear Magnetic Resonance (^1^H NMR)

A ^1^H NMR analysis was performed to evaluate the chemical structure and to determine the Dex oxidation degree.

**Figure 6:**
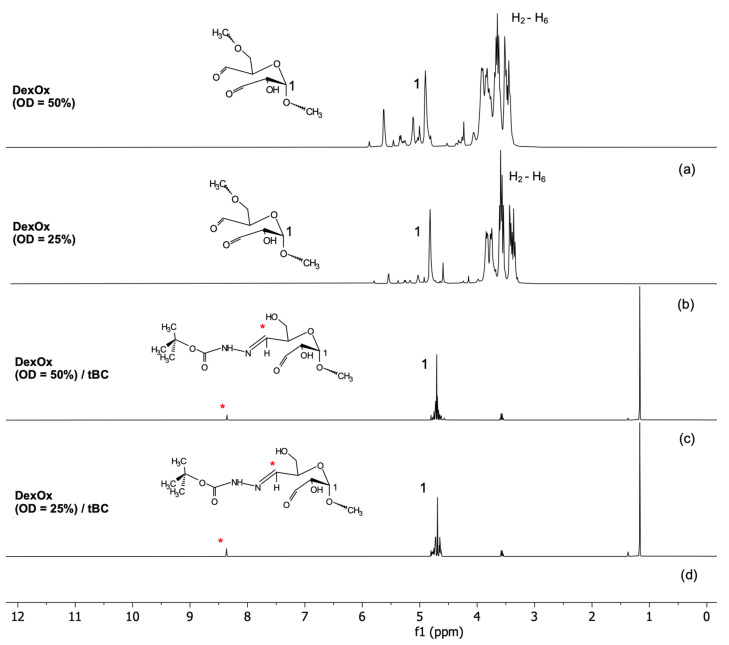
^1^H NMR spectra of (**a**) (OD = 50%), (**b**) DexOx (OD = 25%), (**c**) DexOx (OD = 50%) reacted with tBC, and (**d**) DexOx (OD = 25%) reacted with tBC, with the peaks at 8.3 ppm (*) and 4.8 ppm (1) marked.

**Figure 7:**
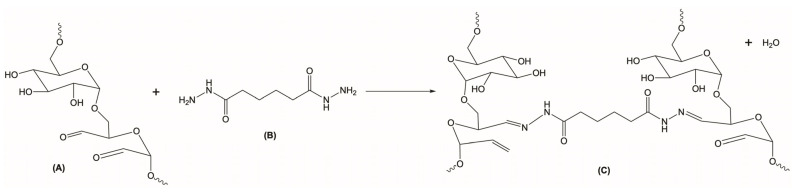
DexOx crosslinking reaction with AAD. (**A**) DexOx. (**B**) AAD. (**C**) Crosslinked DexOx, with hydrazone bonds formation.

Figure 6a,b shows the peaks referring to the hydrogens of the carbons that constitute the glycosidic ring (*H*_2_ to *H*_6_). The peak corresponding to the protons of the aldehyde groups of DexOx should be located at 9.7 ppm; however, this is not visible in the spectrum, which may be related to two factors, the formation of hemiacetals and the temperature at which the reaction occurred. At higher temperatures, more aldehyde groups are formed, which can translate into the appearance of the peak [27]. Moreover, in Figure 7A,B, in the range from 4.2 ppm to 5.8 ppm, the smallest peaks that are observed correspond to the hemiacetals formed during Dex oxidation. In this range, it can be observed that in the DexOx spectrum (OD = 50%), the peaks are more intense than in the DexOx spectrum (OD = 25%). This is due to the presence of more oxidized units, which provides the formation of more hemiacetals. These peaks are not observed in Figure 6c,d since after the addition of tert-butyl carbazate (tBC), the hemiacetals are partially converted into aldehydes, thus allowing the reaction with tBC [15]. The peak corresponding to the anomeric proton of *C_1_* can be seen at 4.8 ppm.

The OD determination is based on the ratio between the integral of two peaks, located at 8.3 ppm and 4.8 ppm, which are represented and marked in the spectra of Figure 6. The peak located at 8.3 ppm concerns the proton bond formed between DexOx and tBC, which is directly related to the Dex oxidation degree [15]. In the literature, this peak is shifted to the right in the spectrum. Through some studies, it is possible to relate these deviations with the conformation of the molecules, since depending on whether they are in the *cis-* or *trans*-form, the peak may appear more to the right or more to the left in the ^1^H NMR spectrum [28]. The peak at 4.8 ppm corresponds to the *C*_1_ proton of the Dex glycosidic ring common in all the Dex glucose units.

Using the software *MestReNova*^©^ version 14.2, the area corresponding to these two peaks was calculated, and through the ratio between them, it was possible to determine the OD of DexOx (OD = 25%) and DexOx (OD = 50%), which resulted at 15.7% and 42.6% oxidation, respectively. Table 2 shows the results obtained experimentally for the OD value, for the first (OD) and second oxidation (OD_sec_) of Dex.

For a theoretical OD of 25%, the experimentally obtained OD value for the first and second oxidations were 15.7% and 7.6%, respectively. For a theoretical OD of 50%, the values obtained were 42.6% and 13.2%, respectively. These values, although lower than expected, are consistent with data from the literature, as can be seen in Table 2. DexOx second OD (OD = 50%) can only be inferred with DexOx (OD = 25%) due to the absence of references in the literature. Therefore, the results obtained experimentally are consistent.

### 3.3. Synthesis of DexOx/AAD Scaffolds

DexOx/AAD scaffolds were prepared by chemical crosslinking. This mechanism is based on the formation of a Schiff base [15,23] through the reaction of the DexOx aldehyde groups with the hydrazide groups of AAD, thus forming hydrazone bonds, as shown in Figure 7.

Both DexOx with an OD = 25% and with an OD = 50% were reacted with AAD as previously described. However, after the predetermined reaction period, it was verified that the hydrogels which resulted from DexOx with an OD = 25% were too fragile to handle and lacked adequate mechanical resistance for the intended purpose. Therefore, the subsequent characterization techniques were applied to the scaffolds obtained with the Dex modified with an OD = 50%.

### 3.4. Swelling Ratio (S_r_)

**Figure 8:**
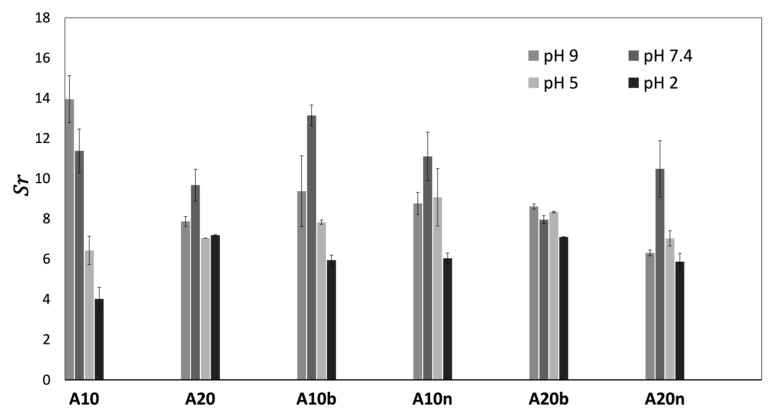
Swelling ratio (*S_r_*) at different pH values for the different composition samples.

Figure 8 shows the obtained results for the swelling ratio (*S_r_*) measured at 24 h of incubation. In general, the swelling capacity is higher at a pH value of 7, close to the physiological pH value (7.4), due to the higher stability of the samples at a neutral pH value. This behavior allows them to remain swollen for higher periods of time, without suffering a rapid degradation that causes the loss of water from their three-dimensional matrix.

In the same way, it is possible to verify that the samples that present lower *S_r_* values are the ones incubated at a pH value of 2. These results are explained by the favored degradation in an acid medium, which means that, as the sample absorbs water (due to the hydrophilic character of the hydroxyl groups) [29], some bonds undergo degradation, which leads to a lower *S_r_*. In general, at pH values of 5 and 9, intermediate *S_r_* values were obtained. Moreover, by analyzing the results of the statistical analysis presented in Table S1, the pH value significantly influences the *S_r_* of the different materials; however, this influence is more evident for A10 samples with or without inorganic compounds. This might be explained by the fact that the increase of AAD leads to a decrease in the swelling capacity, since the increase in crosslinking provides a more closed structure with more crosslinking points. This makes it more difficult for water to enter the structure, despite its hydrophilic character, leading to less significant differences between the *S_r_* results with pH values. However, this tendency was not verified for samples without inorganic compounds at pH values of 5 and 2, and for the samples with β-TCP at a pH value of 2. These exceptions may be related to the fact that at a pH value of 2, the medium is more prone to sample degradation, which causes its faster degradation (as confirmed by the results presented in Section 3.5 Degradation) and, therefore, after 24 h of incubation, the registered swelling may no longer correspond to the maximum swelling. In the next section (Section 3.5. Degradation), a deeper explanation is given concerning the pH environment and the behavior of the hydrogels.

Regarding the physiologic pH results from Figure 8 and analyzing Table S2, the influence of the inorganic compounds in the polymeric structure generally does not follow a trending behavior; in some cases, the samples with β-TCP present a higher *S_r_*, which also happens in some samples with nHAp. Nevertheless, from Table 2, it can be inferred that significant differences are observed for A10, A10b, and A10n, which means that the inorganic compounds have some influence, while these differences are not so significant for the A20, A20b and A20n samples. So, regarding the swelling, the main conclusion which can be made is that the amount of the crosslinker significantly affects the swelling (as previously explained), which may also influence the inorganic incorporation in the matrix of the hydrogel.

### 3.5. Degradation

**Figure 9:**
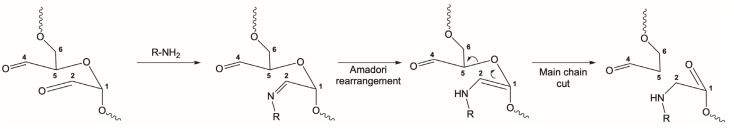
Schematic representation of the Amadori rearrangement and scission of the glycosidic ring.

Before evaluating the results of the in vitro degradation, it is necessary to understand the degradation mechanisms to which these hydrogels are subject. The produced hydrogels have hydrazone linkages in the crosslinking points and glycosidic linkages in the main chain of DexOx. Glycosidic bonds are typically degraded by hydrolysis (favored in acidic medium) [30]. In addition to hydrolysis, according to the literature [29,31], the main chain of DexOx can be degraded by the Maillard reaction, which is triggered by the formation of the Schiff base. These literature data were supported by the ^1^H NMR analyses, where it was verified that the hemiacetal structures that are formed when Dex is oxidized with periodate react with the amine groups and undergo an Amadori rearrangement. In turn, this rearrangement causes ring scission of glycosidic units. Figure 9 shows these mechanisms. There is a possibility that, in the present work, the samples have suffered this type of degradation; however, no difference was detected in the color of the gels, which could indicate its occurrence.

Regarding the hydrazone bonds, they can also undergo degradation by hydrolysis, which corresponds to the reverse reaction of the formation of these bonds. When hydrogels are in the presence of water, the equilibrium of the crosslinking reaction shifts in the opposite direction, causing the breakage of these bonds that constitute the crosslinking points of the hydrogels, leading to their degradation.

**Figure 10:**
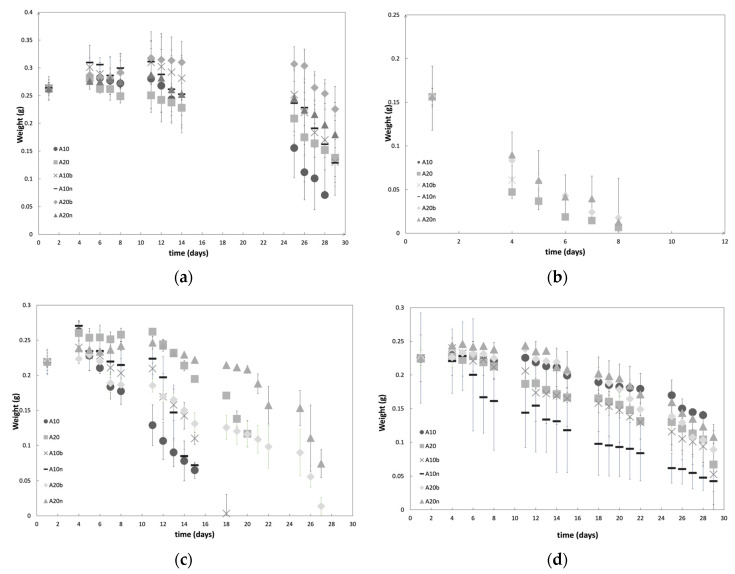
Degradation profiles (mass weight variation as a function of time) of the different samples (**a**) at pH 7.4; (**b**) at pH 2; (**c**) at pH 9; and (**d**) at pH 5.

Degradation studies, in terms of evaluating mass variation over time, were carried out for different compositions and for different pH values and are presented in Figure 10. Figure 10a shows the tendencies obtained for DexOx/AAD hydrogels at a pH value of 7.4, which were the focus, since they correspond to the physiological pH value.

Figure 10a shows that samples with 10% of the crosslinker present a higher rate of degradation. The DexOx samples with 10% AAD degraded after 28 days, while the remaining samples with 10% AAD continued losing mass after 29 days; however it is possible to verify a higher mass loss when compared to the samples with 20% AAD. These results agree with the *Sr* behavior, that is, samples with a lower crosslinker percentage lead to structures with lower crosslinking density, which provides a higher *Sr*. Therefore, the presence of water triggers the degradation of hydrazone bonds, and the lower crosslinking density facilitates the entry of water into the material matrix, which leads to faster degradation. Moreover, samples with 10% AAD showed a mass increase in the first 4/5 days, which can be explained by the fact that, due to the degradation of some crosslinking points, the three-dimensional network becomes more open (with more free space), and this allows the structure to absorb more water, causing an increase in mass.

On the other hand, for the first 4/5 days, samples with 20% AAD do not show such a significant mass increase, since they have a higher crosslinking density; even if the structure starts to degrade due to the presence of water inside, this occurs more slowly, due to the more closed configuration of the network. However, for all samples there is an initial increase in mass in the first 4/5 days, followed by a decrease, and between days 8 and 11 there is again an increase in mass. These oscillations lead to the assumption that in the first ten days, degradation of the structure occurs by hydrolysis, and simultaneously, water absorption occurs while the structure still has a relatively dense three-dimensional network. It should be noted that, in addition to the degradation of the hydrazone bonds, the degradation of the glycosidic bonds also occurs, thus breaking the main chains of DexOx. The breaking of glycosidic bonds has the particularity of forming more hydroxyl groups, which contributes to the absorption of more water in the initial period of degradation. After 10/11 days of degradation, there is a decrease in the mass, without oscillations, since the structure of the material is already compromised.

As for the presence of inorganic compounds in the material, by analyzing the degradation profiles, a slightly slower degradation profile can be seen, presenting a smaller mass loss. Generally, at a pH value of 7.4, the hydrogels have a slow degradation of about one month, which, for the intended application, is very promising.

In Figure 10b, the degradation profiles at a pH value of 2 are represented; these profiles are distinct from those at a pH value of 7.4, since it takes only 8 days for the samples to be fully degraded. This behavior was expected, considering that the samples are subject to a more aggressive environment. At acidic pH values, there is a higher amount of H_3_O^+^ ions, which favors the hydrolysis of the hydrazone bonds and glycosidic bonds, which in turn causes a much faster degradation [15]. The samples with 10% AAD, once again, degraded faster than the others (after 1 day), due to the same reasons mentioned previously, but which, in an acidic environment, are much more notorious. Regarding the influence of the two inorganic compounds, once again there were no significant differences between their influence.

Figure 10c shows the degradation profiles at a pH value of 9, where there is a significant difference between the degradation time of the samples with 10% and 20% AAD. This might be due to the fact that, with a smaller amount of the crosslinker, there is a lower degree of crosslinking, and therefore higher swelling; in turn, this leads to faster degradation. Within the samples with 10% AAD, the samples without inorganic compounds were the first to degrade, which leads to the assumption that the presence of inorganic compounds strengthens the structure, influencing, in general, the degradation of the hydrogel.

Finally, Figure 10d shows the degradation profiles of the samples at a pH value of 5. The profiles obtained for the hydrogels at a pH value of 5 are similar to the profiles obtained at a pH value of 7.4; that is, the hydrogels keep most of their integrity during the period of 29 days. Regarding the amount of crosslinker, the hydrogels once again show the same behavior that was described previously.

In general, the degradation profiles at a pH value of 7.4 are the focus, since under normal conditions, the material will be at the physiological pH value. With the addition of the drug to the material (which will be described later), a pH variation may occur in the area surrounding the material, since the drug solution has a more acidic pH value (2.5–4.5) [32]. Furthermore, if there is any kind of infection at the site, it is likely that the pH value will decrease. Thus, it is also interesting to focus attention on the profiles obtained at a pH value of 5. According to the results of the degradation at a pH value of 5, the material degrades in approximately one month, just as it does at a pH value of 7.4. These results are advantageous in that neither the introduction of the drug nor a possible infection will significantly compromise the durability of the material, even with a decrease in the pH value caused by these situations.

**Figure 11:**
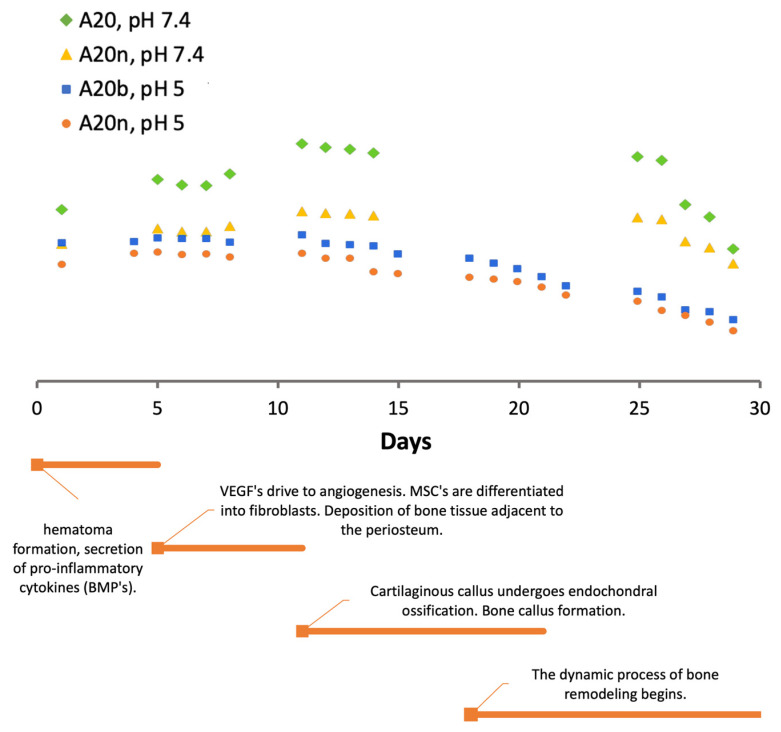
Timeline of the bone healing/regeneration process.

Figure 11 shows the degradation profiles obtained at pH values of 7.4 and 5 in parallel with the timeline of the bone healing/regeneration process. The samples chosen for this analysis were the samples with 20% AAD and with the presence of inorganic compounds, since they showed the best results.

Through the results presented in Figure 11, it can be verified that in the first 5 days, when the formation of hematoma and the release of BMPs occurs, the swelling of the material also occurs, which means that the material contains a high amount of fluids in its interior. This swelling is favorable at this stage, as it facilitates the mobility of fluids that direct BMPs to the site, as well as cells and nutrients. The same happens in the second phase, during days 5–10, when VEGFs and MSCs migrate to initiate angiogenesis and differentiation into fibroblasts. Up to approximately the 12th day, the material absorbs a significant amount of fluids as it degrades. This swelling is more noticeable in profiles at a pH value of 7.4, which is the ideal pH condition. At a pH value of 5, the swelling is not so significant, which may translate into a slightly difficult mobility of the fluids and bioactive substances involved. Analyzing the third and fourth phases, where the endochondral ossification of the cartilaginous callus and formation of bone callus begins, the degradation profile shows a decrease until the end of the 30 days. This behavior suggests that as bone deposition occurs, the material undergoes degradation, allowing the formation of new tissue.

It should be noted that these results are beneficial and meet the intended objective; however, the bone healing/regeneration process in older individuals occurs slowly. Thus, it would be advantageous for the degradation of the material to be longer to ensure that it complements the regeneration process until the end.

### 3.6. Compression Tests

Compression tests were performed, and the obtained Young’s Modulus and the maximum compressive stress values of all materials are shown in Table 3.

As can be seen in Table 3, the samples presented Young’s modulus values between 200 and 300 kPa. Analyzing the differences between the various compositions, it is possible to verify that by increasing the crosslinking percentage, the samples become more resistant, which is translated into a higher Young’s modulus. The main objective of introducing inorganic elements into the matrix was to reinforce mechanical properties. This effect was demonstrated, since for both samples (10% and 20% AAD), the introduction of an inorganic compound led to a higher Young’s modulus value.

No significant differences were observed between the two inorganic materials, which leads to the conclusion that the use of these inorganic compounds increases the mechanical properties of the supporting matrix, but there is no preference between them regarding the reinforcement of the material.

In the literature, a wide range of Young’s modulus values for this type of three-dimensional support matrix can be found. Hydrogels based on Dex (similar to those described in the present work) present values of approximately 140 ± 29 kPa [16], slightly lower than those obtained in the hydrogels developed in this study. In another study using PEGDM (poly(ethylene glycol) dimethacrylate) hydrogels reinforced with NFC (cellulose nanofibers), Young’s modulus values in the range of 150–300 kPa were obtained, depending on the amount of cellulose incorporated [28]. These results are more in line with the results obtained in this work. In another study that developed cryogels based on dextran and gelatin, lower Young’s modulus values of 2.78 ± 0.08 kPa were obtained [29]. Finally, a study with hydrogels based on collagen, PCLC (poly(lactide-co-ε-caprolactone), and hydroxyapatite, reported a Young’s modulus value of 5 kPa [4]. Therefore, the values obtained for the Young’s modulus of the studied materials are quite favorable for bone regeneration processes (infections occur during implantation time, bone graft substitutes with antibiotics), considering the overview of the values found in the literature for similar materials [33,34].

### 3.7. Drug Release

**Figure 12:**
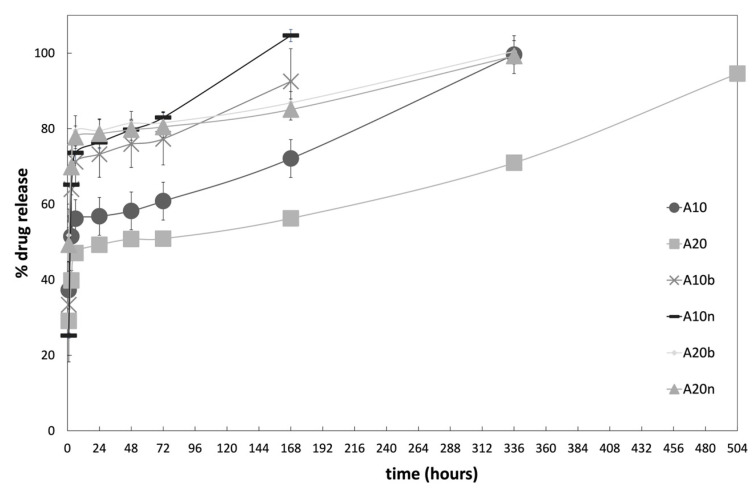
Cumulative controlled vancomycin release profiles for the different hydrogels.

The controlled release of vancomycin was carried out for the different compositions of the hydrogels and the cumulative release profiles obtained are shown in Figure 12.

Analyzing the cumulative curves, it is possible to verify that there is an initial burst during the first 6 h, reaching more than 50% of drug release, followed by a much slower phase. This initial behavior is advantageous for applications in bone defects and fractures, as the rapid release during the initial stage will prevent possible infections by causing the death of undesirable microorganisms present at the implant site, and by preventing the formation of biofilms. The next phase of slower release prevents infections at the implant site during the process of healing and regeneration of tissues [30].

**Figure 13:**
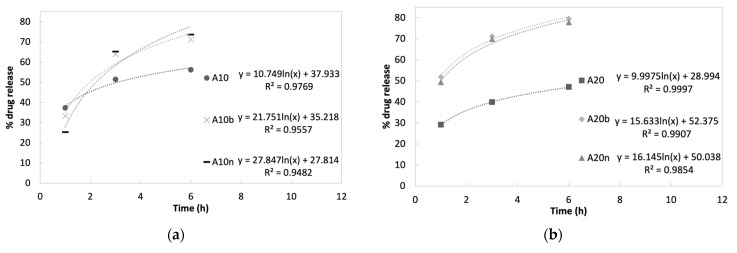
Cumulative controlled vancomycin release profiles during the first 6 h for (**a**) hydrogels with 10% AAD and (**b**) hydrogels with 20% AAD.

In Figure 13, it can be observed that the vancomycin release profile during the initial 6 h burst follows a logarithmic rate for all samples, with high determination coefficients (R^2^ > 0.985). These profiles are associated with the release of the drug’s molecules located on the surface of the support matrix, and mainly due to the diffusion phenomenon of the drug from the matrix to the surrounding moiety. The equation coefficients of the logarithmic trendlines allow us to quantify the different profiles’ release rates. There is no significant difference between the hydrogels without inorganic compounds since 10% and 20% AAD samples have similar release rates: 10.749 and 9.9975, respectively. The presence of either β-TCP or nHAp increases the release coefficient, especially in the 10% AAD samples, where the coefficient doubled for the β-TCP samples (21.751) and almost triplicated for the nHAp samples (27.847). This drug release increase is beneficial to the prevention of infection, as high amounts of vancomycin will assure the elimination of any pathogens already present on the bone’s surface at the time of scaffold implantation.

**Figure 14:**
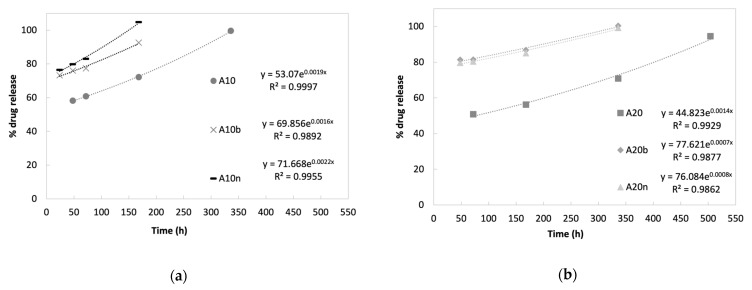
Cumulative controlled vancomycin release profiles during the last stage of the study for (**a**) hydrogels with 10% AAD and (**b**) hydrogels with 20% AAD.

The final stage of drug release is related to the molecules trapped inside the matrix, where release is dependent on the material’s degradation, providing a slower release [30]. This is evident by the fact that the release profiles follow an exponential rate. With the degradation of the samples following a constant rate during the last two weeks of the study (see Section 3.5. Degradation), the surface/mass ratio of the samples increases exponentially, leading to an exponential release of the drug that was entrapped in the matrix. Figure 14 depicts the release profiles during the last stage, with the exponential trendlines and equations associated with each drug release profile. The high values of determination coefficients (R^2^ > 0.986) demonstrate the excellent fit between the experimental data and the model.

As expected, the exponent factor is correlated with the degradation rate observed during the last weeks of the study period—the higher the degradation rate, the higher the exponent factor. For instance, the 10% AAD samples, either without inorganic compounds or with nHAp, which showed the fastest degradation rates, present an exponent factor of 0.0019 and 0.0022. On the other hand, the samples with 20% AAD with either inorganic compound release the drugs slowly, with exponent factors of 0.0008 for nHAp, and 0.0007 for β-TCP.

Most profiles demonstrate that it is possible to maintain a drug release for about 14 days, which is quite favorable, considering that, under normal circumstances, on the 5th day of the bone healing/regeneration process, the deposition of bone tissue begins. This means that the phase with the greatest probability of infection would take place in the presence of an antibiotic. It should be noted that only the samples with 20% AAD and without inorganic compounds present a release of up to 21 days, unlike the others. These results result from a higher crosslinking degree, which in turn causes slower degradation, which leads to a lower release rate.

Emphasizing the influence of inorganic compounds on the release profiles, it is clear that the samples with these materials in their composition present a faster drug release. In the case of samples with inorganic compounds and 10% AAD, the release occurred only for 7 days, and with an initial burst reaching around 70% to 80% of the drug. On the other hand, samples with inorganic compounds and 20% AAD presented the same behavior concerning the initial burst, but with a drug release lasting for 14 days. These results indicate that the drug release rate is influenced both by the degree of the crosslinking of the samples and by the presence of inorganic compounds in the matrix. The higher the degree of crosslinking, the slower the drug release, as expected. The presence of inorganic compounds led to a faster release, which may be associated with the possible increase in porosity caused by the addition of these compounds, inorganic and drug, to the material matrix. Their presence might induce more pores in the matrix of the scaffold, which in turn would allow the entry and circulation of fluids, causing an increase in the diffusion of the drug out of the material.

The amount of drug present in each sample is slightly low, as the initial solution of vancomycin for the injection was diluted according to the manufacturer’s instructions, resulting in a low concentration solution (5% (*w*/*v*)). However, it is important to check the recommended doses for the use of this drug. Table 4 summarizes the recommended dosages for treatment with intravenous vancomycin [31].

For the intended application of this work, the action of the drug will be localized, meaning that it will be placed exactly at the target site; therefore, the amount of drug necessary to prevent infections will be lower than the recommended doses for intravenous administration to treat an existing infection (Table 4).

As an example, considering the samples with 20% AAD and the incorporated inorganic compounds, a release of about 80% during the first 6 h was obtained, which renders into 10 mg of vancomycin. Considering the values in Table 4, and that the goal is to prevent infections by acting directly on the site and not through the systemic route, we can infer that the amount of the drug that is present in the samples is enough and could even be lower.

### 3.8. Biocompatibility

**Figure 15:**
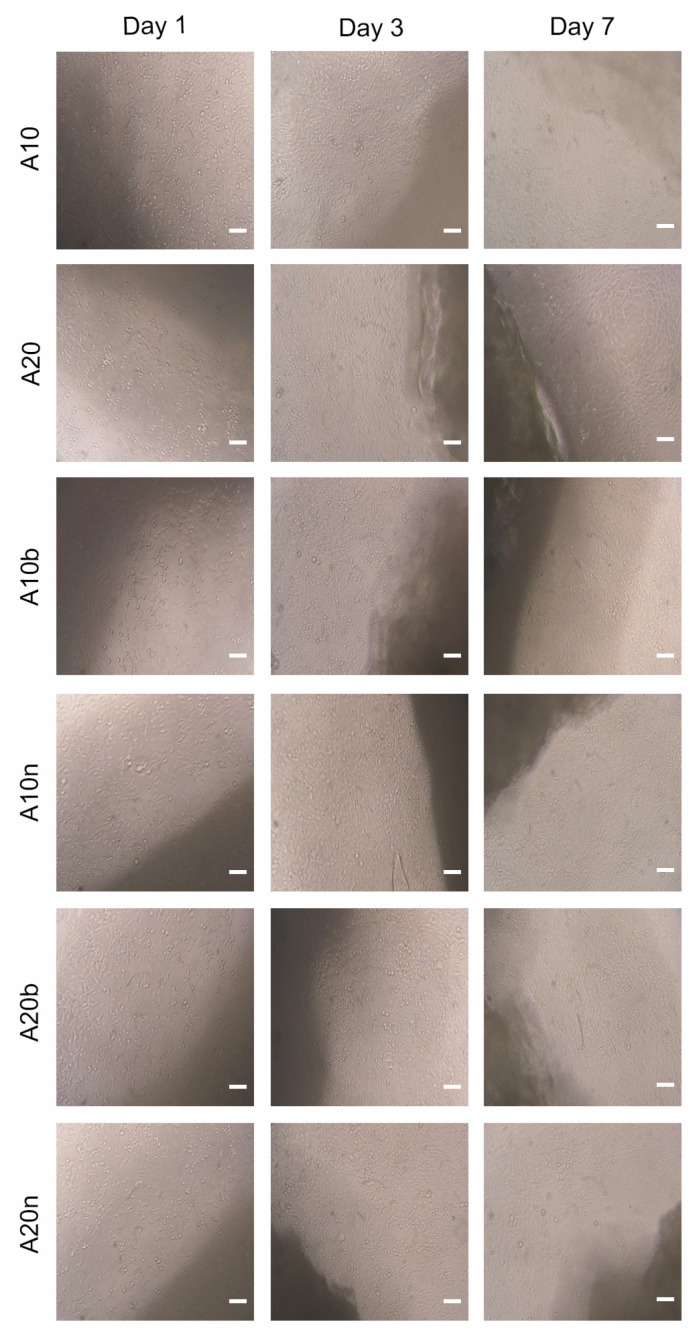
Optical microscopic images of hOB cells cultured in contact with the produced hydrogels after 1, 3, and 7 days of incubation. Scale bars correspond to 100 μm.

**Figure 16:**
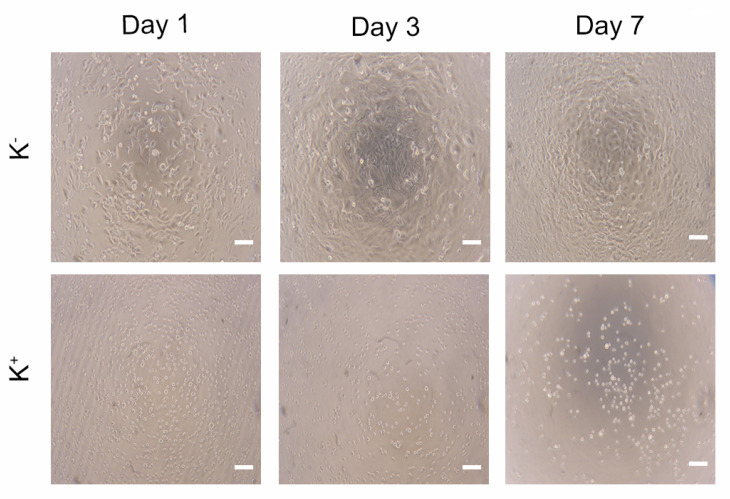
Optical microscopic images of hOB cells in the negative control (K^−^, cells incubated with culture medium) and positive control (K^+^, cells incubated with EtOH 70%) after 1, 3, and 7 days of incubation. Scale bars correspond to 100 μm.

The cytocompatibility of the different hydrogels was evaluated using hOB cells (as cell model), through MTS assay. To accomplish the characterization of the biological properties of the different hydrogels, hOB cells were seeded in contact with the produced hydrogels and then optical microscopic images were acquired (Figure 15) to characterize the morphology of the cells. The images show that the hOB cells exhibited an elongated morphology like those cells present in the negative control (i.e., cells incubated with culture medium only), in the first days. However, after 7 days of contact, the images show some cells with a spherical shape, similar to the cells present in the positive control (characteristic of dead cells). The negative and positive controls after 1, 3, and 7 days are presented in Figure 16.

**Figure 17:**
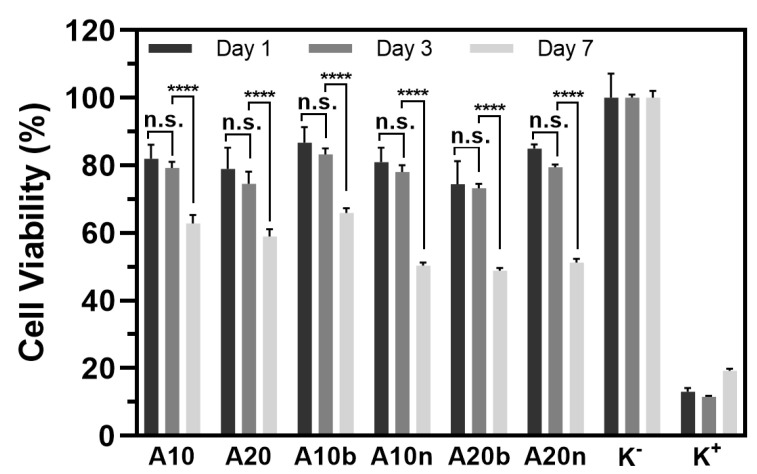
Characterization of the hydrogels’ biocompatibility. Evaluation of the viability of hOB cells when cultured in the presence of the produced hydrogels after 1, 3, and 7 days of incubation, through MTS analysis. K^−^ (live cells); K^+^ (dead cells). Data are presented as the mean ± standard deviation, n = 5, **** *p* < 0.0001, n.s. = non-significant.

In addition, the results of the MTS assay, as can be seen in Figure 17, show that after 1 and 3 days of incubation with the samples, the cells presented cell viabilities superior to 70%, demonstrating that the cells remain viable. However, the results on day 7 showed a decrease of the viabilities to lower than 70%, which was not intended. These results may be related to the presence of AAD and are in agreement with some works in the literature [35,36]. For example, Zhang et al. produced scaffolds based on collagen and hyaluronic acid modified with AAD and compared its biocompatibility with those without AAD. The authors observed that scaffolds with AAD presented higher biocompatibility on days 2 and 4; however, on day 6, those with AAD showed a relatively slower cell growth rate [36].

Moreover, analyzing the results of the incorporation of the different inorganic compounds, the samples with 10% AAD and β-TCP presented higher biocompatibility in comparison with the samples with 10% AAD and nHAp, suggesting that β-TCP would be better for cell viability. However, in the samples with 20% AAD, this difference was not verified; i.e., in this case, samples with 20% AAD and nHAp showed higher biocompatibility than those with β-TCP. Therefore, it can be stated that both the amount of the crosslinker and the type of the inorganic compound have an influence on the biocompatibility of the material.

In general, the results are satisfactory for the first few days; however, the cytotoxicity that the material showed on day 7 suggests that the amount of AAD, or its replacement, should be rethought and reassessed.

## 4. Conclusions

The results showed that the synthesis of dextran-based hydrogels was successful. The synthesis process of the hydrogels involved the previous step of modifying dextran. Through ATR-FTIR and ^1^H NMR analyses, this modification was successfully proven, and the OD determination was as expected: DexOx with an OD of 14.8% for the first oxidation, and 7.6% for the second oxidation, was obtained. After the synthesis of the hydrogels and their chemical characterization, several parameters were evaluated, such as degradation and vancomycin release; further, compression tests and biocompatibility tests were performed.

Regarding the degradation of the hydrogels, which was tested at four different pH values, in general, the samples at pH values of 2 and 9 had a shorter shelf life, as expected. At pH values of 7.4 and 5, which would be the most likely values in the environment in which these samples will be exposed during their application, they lasted about a month until they degrade, which corresponds to the regeneration time of bone tissue in normal circumstances. In general, the material presented an adequate degradation rate, which will match the rate of bone regeneration. Of all the compositions tested, the samples with a higher amount of crosslinker and with the presence of inorganic compounds (A20b and A20n) showed better results, as they have a higher crosslinking density, which translates into slower degradation.

Compression test results were relatively consistent with data found in the literature for this type of materials. The Young’s modulus presented values between 200–300 kPa. Samples with the highest modulus values, i.e., more resistant, were again the ones with a higher AAD percentage and with inorganic compounds (A20b and A20n), which was expected.

The drug release lasted an average of 14 days; however, the release profile showed an initial burst release of around 50–80%. This makes longstanding treatment difficult, but it can facilitate the prevention of infections in the initial period. Regarding the material’s biocompatibility, the results showed that by the 3rd day, the cells had a viability of around 70 to 80%. On the 7th day, the results obtained showed a more significant decrease, around 50 to 60% of cell viability. This behavior could be associated with AAD, which, due to its acidic nature, may present a mild inhibitory effect on cell proliferation.

## Figures and Tables

**Table 1:** Hydrogels’ composition.

Hydrogel	DexOx (OD = 50%) % (*m*/*v*)	AAD %	β-TCP % (*m*/*v*)	nHAp % (*m*/*v*)
A10	20	10	-	-
A20		20	-	-
A10b		10	1	-
A10n		10	-	1
A20b		20	1	
A20n		20	-	1

**Table 2:** Dex oxidation degree (OD), theoretical (th) and experimental (exp).

${OD}_{th}$ (%) ^1^	${OD}_{exp}$ (%) ^2^	${OD}_{lit}$ (%)	${OD}_{sec,exp}$ (%) ^3^	${OD}_{sec,lit}$ (%)
25	15.7	14.3 [15]	7.6 ± 0.4	7.1 ± 0.1 [15]
50	42.6	43 [16]	13.2	-

^1^ Determined as the molar ratio of sodium periodate to initial units of glucose. ^2^ Determined by ^1^H NMR analysis. ^3^ Determined by formic acid titration.

**Table 3:** Young’s modulus and maximum compressive stress results obtained for each hydrogel.

Hydrogel	Young’s Modulus (kPa)
A10	206 ± 33
A20	250 ± 39
A10b	240 ± 24
A10n	243 ± 18
A20b	283 ± 77
A20n	277 ± 40

**Table 4:** Recommended dosages for treatment with intravenous vancomycin.

	Recommended Therapeutic Dosage	Maximum Dose
≥12 years old	$15\;\mathrm{to}\;20\;{mg/kg\;}_{body\;weight}$ every 8 to 12 h	Do not exceed 2 g
Infants and children aged 1 month to 12 years	$10\;\mathrm{to}\;15\;{mg/kg\;}_{body\;weight}$ every 6 h	_

## Data Availability

The raw/processed data required to reproduce these findings cannot be shared at this time due to technical or time limitations but will be sent upon request.

## Supplementary Materials

The following supporting information can be downloaded at: https://www.mdpi.com/article/10.3390/polym15234501/s1, Figure S1: Image of the obtained hydrogels with different compositions; Table S1: Statistical analysis of the swelling results. * *p* < 0.1, ** *p* < 0.01, *** *p* < 0.001, **** *p* < 0.0001, n.s. = non-significant. Table S2. Statistical analysis of the swelling results at pH 7.4. * *p* < 0.1, ** *p* < 0.01, *** *p* < 0.001, **** *p* < 0.0001, n.s. = non significant.
