# Supplementary material for: Dextran-Based Injectable Hydrogel Composites for Bone Regeneration
Source: Polymers (Basel). 2023 Nov 23;15(23):4501. doi: 10.3390/polym15234501 (PMC10707775; doi:10.3390/polym15234501)
Supplement: Supplementary file 1 [file polymers-15-04501-s001.zip › polymers-2648938-supplementary.pdf]

Supplementary Information

Dextran-based injectable hydrogel composites for bone regeneration

Patrícia Alves [1,*], Ana Filipa Simão [1], Mariana F.P. Graça [2], Marcos J. Mariz [1], Ilídio J. Correia [1,2] and Paula Ferreira [1,3]

[1] University of Coimbra, CIEPQPF, Department of Chemical Engineering, Faculty of Sciences and Technology, Rua Sílvio Lima, 3030-790 Coimbra, Portugal; palves@eq.uc.pt (P.A.), gouveia.fii@gmail.com (A.F.S.) mmariz@eq.uc.pt (M.J.M.)
[2] CICS-UBI, Health Sciences Research Centre, Faculty of Health Sciences, University of Beira Interior, 6200-506 Covilhã, Portugal; marianaf_g@hotmail.com (M.F.P.G.), icorreia@fcsaude.ubi.pt (I.J.C.)
[3] Polytechnic Institute of Coimbra, Applied Research Institute, Rua da Misericórdia, Lagar dos Cortiços – S. Martinho do Bispo, 3045-093 Coimbra, Portugal; paula.ferreira@ipc.pt (P.F.)
* Correspondence: palves@eq.uc.pt (P.A.)

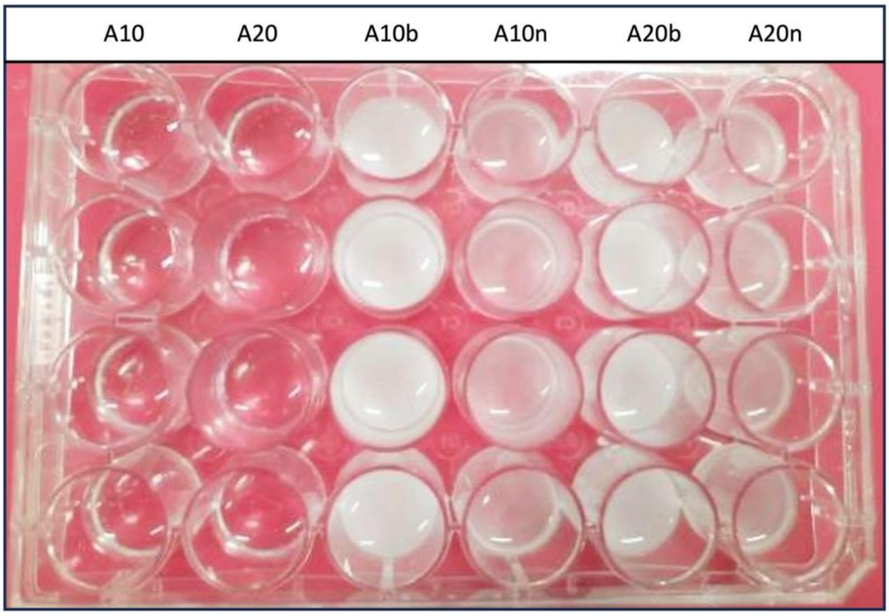

Figure S1. Image of the obtained hydrogels with different compositions.

Table S1. Statistical analysis of the swelling results. *p< 0.1, **p< 0.01, ***p< 0.001, ****p< 0.0001, n.s. = non significant.

Multiple group comparisons	Statistical analysis
A10 - pH 7,4 vs A10 - pH 5	****
A10 - pH 7.4 vs A10 - pH 9	***
A10 - pH 7.4 vs A10 - pH 2	****
A10 - pH 5 vs A10 - pH 9	****
A10 - pH 5 vs A10 - pH 2	**
A10 - pH 9 vs A10 - pH 2	****
A20 - pH 7,4 vs A20 - pH 5	**
A20 - pH 7.4 vs A20 - pH 9	n.s.
A20 - pH 7.4 vs A20 - pH 2	**
A20 - pH 5 vs A20 - pH 9	n.s.
A20 - pH 5 vs A20 - pH 2	n.s.
A20 - pH 9 vs A20 - pH 2	n.s.
A10b - pH 7,4 vs A10b - pH 5	****
A10b - pH 7.4 vs A10b - pH 9	****
A10b - pH 7.4 vs A10b - pH 2	****
A10b - pH 5 vs A10b - pH 9	n.s.
A10b - pH 5 vs A10b - pH 2	n.s.
A10b - pH 9 vs A10b - pH 2	***
A10n - pH 7,4 vs A10n - pH 5	*
A10n - pH 7.4 vs A10n - pH 9	**
A10n - pH 7.4 vs A10n - pH 2	****
A10n - pH 5 vs A10n - pH 9	n.s.
A10n - pH 5 vs A10n - pH 2	***
A10n - pH 9 vs A10n - pH 2	**
A20b - pH 7,4 vs A20b - pH 5	n.s.
A20b - pH 7.4 vs A20b - pH 9	n.s.
A20b - pH 7.4 vs A20b - pH 2	n.s.
A20b - pH 5 vs A20b - pH 9	n.s.
A20b - pH 5 vs A20b - pH 2	n.s.
A20b - pH 9 vs A20b - pH 2	n.s.
A20n - pH 7,4 vs A20n - pH 5	****
A20n - pH 7.4 vs A20n - pH 9	****
A20n - pH 7.4 vs A20n - pH 2	****
A20n - pH 5 vs A20n - pH 9	n.s.
A20n - pH 5 vs A20n - pH 2	n.s.
A20n - pH 9 vs A20n - pH 2	n.s.

Table S2. Statistical analysis of the swelling results at pH 7.4. *p< 0.1, **p< 0.01, ***p< 0.001, ****p< 0.0001, n.s. = non significant.

**Multiple group comparisons**	**Statistical analysis**
A10_ pH 7.4 vs. A20_ pH 7,4	*
A10_ pH 7.4 vs. A10b_ pH 7,4	**
A10_ pH 7.4 vs. A10n_ pH 7,4	ns
A20_ pH 7,4 vs. A20b_ pH 7,4	ns
A20_ pH 7,4 vs. A20n_ pH 7,4	ns
A10b_ pH 7,4 vs. A20b_ pH 7,4	****
A10n_ pH 7,4 vs. A20n_ pH 7,4	ns
A10b_ pH 7,4 vs. A10n_ pH 7,4	**
A20b_ pH 7,4 vs. A20n_ pH 7,4	**
